# Importance of New Particle Formation for Climate and Air Quality

**DOI:** 10.1021/acsestair.5c00095

**Published:** 2025-04-16

**Authors:** Markku Kulmala

**Affiliations:** † Institute for Atmospheric and Earth System Research/Physics, Faculty of Science, University of Helsinki, Helsinki 00014, Finland; ‡ Aerosol and Haze Laboratory, Beijing Advanced Innovation Center for Soft Matter Science and Engineering, Beijing University of Chemical Technology, Beijing 100029, China; § Nanjing-Helsinki Institute in Atmospheric and Earth System Sciences, Nanjing University, Nanjing 210023, China; ∥ Joint International Research Laboratory of Atmospheric and Earth System Sciences, School of Atmospheric Sciences, Nanjing University, Nanjing 210023, China

Before the Chernobyl nuclear power plant accident, in Aprill 1986, there was some but rare knowledge of atmospheric new particle formation.^1^ After the accident, we started to develop the SMEAR concept,^2^ one of the cornerstones of conducting continuous and comprehensive measurements. One of our early results was direct observations of atmospheric new particle formation.^3^ Subsequently, we also managed to improve our theoretical understanding of new particle formation and to determine theoretically the atmospheric clusters,^4^ which we later confirmed experimentally.^5^ Although the general understanding of atmospheric new particle formation has improved significantly during the past 40 years,^1,6^ many open questions remain. Here we present a summary of the progress obtained during the past 40 years (1985–2005) and discuss open questions and future research needs.

In 1986, Prof. Seinfeld published his textbook describing the basis for atmospheric chemistry.^7^ At that time, our understanding of atmospheric new particle formation was very limited. The progress in 40 years has been remarkable. The situation and developments are summarized in Table 1. However, the numbers given here are educated estimates based on observational studies and conceptual thinking and need to be carefully examined in future investigations, since there are not yet enough observations around the world to confirm these estimates.

**Table 1:** Global Importance of New Particle Formation (including traffic+) Based on the Knowledge We Had in 1985 and at Present^a^

		1985	2025
aerosol number load		negligible	60–90%
aerosol mass	gas-to-particle conversion	negligible, some contributions	70–95%
CCN load		negligible	50–65%
contribution to haze particle number concentration	contribution to air pollution in megacities	negligible	70–90%
effect on carbon sink (local)	CarbonSink+	no idea	enhacing by 5–80% the climate effects of carbon sink

a The contribution of atmospheric clusters, NPF, and subsequent growth as well as GTP (gas-to-particle conversion) in percent.

During the past 40 years, the measurement technology has improved significantly. Today we are able to detect and chemically identify clusters containing only a few molecules and, at the same time, determine the number concentration and even the size distribution of these clusters (<2 nm in size) in different atmospheric environments,^1^ which was not possible 40 years ago. Furthermore, we started to make comprehensive observations and developed the SMEAR concept, aiming to use it globally^8^ in order to gather comprehensive data from around the world. The global importance cannot be quantified without such global observations.

Traditionally, the aerosol number load is divided into primary and secondary particles, where the total number of aerosol particles is given by
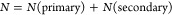
1However, in the case of very small particles and atmospheric clusters, it is often difficult or even impossible to tell whether these particles have been directly emitted to the atmosphere (e.g., from pipelines of cars or power plants) or they are airborne after the immediate cooling of gas plumes. In urban environments, this is particularly true for traffic emissions. Therefore, we should calculate the total number concentration using the expression
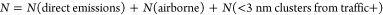
2where traffic+ means power plants and all similar sources where it is unclear if the clusters are directly emitted or airborne. The particle number concentrations are size segregated in observations, and we should, in the future, find ways to define all three categories from observations.

Recent observations show further that particle growth rates (GRs) tend to vary quite little both within and between different environments, much less than the condensable vapor concentration does.^9^ This makes it easier to calculate particle formation rates at desired sizes, provided that the corresponding formation rates at 3 or 5 nm, together with the typical condensation sink (CS) in this area, are known.^10^ However, the reason for this GR mystery is still unknown and requires future investigations.

One example of recent improvements and the usage of comprehensive long-term data is the comparison between observations made in Beijing (Aerosol and Haze Laboratory in Beijing University of Chemical Technology (AHL/BUCT), China) and at the SMEAR II station in Hyytiälä, Finland. The AHL/BUCT station is located in a megacity, and the SMEAR II station in a boreal forest. From Beijing, we have 6 years of comprehensive observations and from Hyytiälä 25 years.^11^ Both locations have more than 1200 simultaneously observed variables. We have utilized time over land/time over populated land (TOL/TOPL) methods together with a recentley developed ranking method for NPF observations.^9^ This method has proven to be very efficient. Furthermore, we have found that we can use 2.0–2.3 nm negative ions as an indicator for local NPF. The formation rates of new particles vary substantially, being about 100–1000 times higher in Beijing than in Hyytiälä. The dominating NPF mechanism in Hyytiälä is sulfuric acid (SA) and organics (and NH_3_), while in Beijing, it is SA_dimer_ and amines (and NH_3_). However, in both places, particle growth rates are very similar.^11^ The total particle mass and number concentrations and the concentrations of most gaseous pollutants (excluding ozone) are much higher in Beijing than in Hyytiälä.

Much has been done in the past 40 years, but has this been sufficient? We have still open questions, such as proving or disproving the numbers in Table 1. Also, it is important to determine why particle growth rates vary so little and the size-segregated composition of aerosol particles. We need to understand the formation and subsequent growth of aerosol particles and their origin to predict the future climate, air quality, health effects, and influences on the water cycle. The contribution of NPF and gas-to-particle conversion (GTP) in general are crucial to global and environmental challenges.

Therefore, we need to have a research program to meet these inherent needs. We probably also need to change our thinking to answer the remaining questions. However, always when we want to change something, we need to go over resistance (*R*) to change:
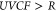
3Therefore, we need to have clear understanding (*U*) and vision (*V*), capacity (*C*) to change, and feasible (*F*) plans to face these inherent issues.

In the future, we will need to continue developing atmospheric mass spectrometry technology to determine the composition and concentrations of gases, vapors, clusters, and aerosol particles. Furthermore, ion and aerosol spectrometers as well as ion counters need to be improved and used in different environments. The need for SMEAR-type observatories in different ecosystems, including megacities, is evident.^8^ However, this is not enough. Remote sensing and particularly satellite data and improving proxies (e.g., vapor, cluster, and aerosol concentrations) for global coverage are needed to determine the global importance of NPF. The theoretical understanding of NPF needs to be improved to be able to develop more reliable models, from process models to regional air quality models and global Earth system models. Finally, the models need to be compared with and validated by observations. Once we are able to reproduce the present and previous observations with existing models, there is some hope of predicting the future behavior of NPF and GTP and, furthermore, of predicting the future climate, haze, clouds, precipitation, and water cycle.
